# Editorial: C1q: A Molecular Bridge to Innate and Adaptive Immunity

**DOI:** 10.3389/fimmu.2020.00417

**Published:** 2020-03-17

**Authors:** Uday Kishore, Berhane Ghebrehiwet

**Affiliations:** ^1^Biosciences, College of Health and Life Sciences, Brunel University London, Uxbridge, United Kingdom; ^2^Department of Medicine, State University of New York, New York, NY, United States

**Keywords:** complement, classical pathway, C1q, C1 complex, infection, cancer, non-complement functions

Human C1q is the first recognition molecule of the classical pathway of the complement system. C1q has a characteristic tulip-like overall structure where N-terminal collagenous stalk (CLR) is followed by a heterotrimeric C-terminal globular (gC1q) domain (1). After recognizing IgG- and IgM-containing immune complexes, C1q, in association with C1r and C1s complexes that yields C1 molecule, triggers the classical pathway activation. However, C1q is not always dependent on the binding of IgG or IgM to target ligands, primarily pathogens, in order to perform its duty as a potent innate immune molecule. In addition to being the key molecule of the classical pathway, C1q has a broad range of functions that includes clearance of apoptotic and necrotic cells, sustenance of pregnancy, recognition of pathogens in an antibody-independent manner, immune cell modulation, and pruning of excess synapse during development (2, 3). The importance of C1q in human health has been highlighted by its involvement in a number of pathological conditions including lupus nephritis, a number of inflammatory disorders, Alzheimer's disease, prion disease, and cancer.

During the course of last two decades, a number of proteins of diverse origin have been shown to contain at least gC1q-like domain, and hence, identification of a C1q family (4). The structural analysis of one of such C1q family member, adiponectin, revealed that the three dimensional structure of gC1q domain was remarkably conserved and overlapped with tumor necrosis factor (TNF) and related molecules, hence, recognition of a C1q-TNF superfamily (2, 5–7).

Although the main source of the local synthesis of C1q has been attributed to the potent antigen presenting cells such as monocyte/macrophages and dendritic cells, various types of proliferating and non-proliferating cells including malignant cells can also be included in the list of C1q producers (8). Thus, there are a number of functions assigned to C1q that are not reliant on complement activation mediated by C1q (non-complement functions of C1q) (3).

In this volume, which highlights the structural and functional complexities of human C1q, Reid, one of the pioneers in the field, has elegantly provided a historical account of the field, together with many unanswered questions and what the future holds for this truly remarkable complement protein. Another review by Lu and Kishore examines important features of C1 complex that can perform exciting and unexpected functions without involving complement activation (9). For instance, C1q, when bound to the Frizzled receptors, leads to activation of C1s, which cleaves lipoprotein receptor-related protein (LRP) 6 to trigger aging-associated Wnt receptor signaling (10). C1q binds to apoptotic cells and the activated C1 proteases cleave nuclear antigens (11). The diversity of C1q ligands and C1 protease substrates makes C1q as well as C1 complex quite a versatile recognition and effector machinery beyond the territory of complement activation. In the continuing theme of structural and functional versatility of C1q, Ghebrehiwet et al. emphasize the modular organization of the gC1q domain, revealing the ‘secret of this functional diversity', based on the modularity within each chain of gC1q domain i.e., ghA, ghB, and ghC modules (12). Within the gC1q domain, which is composed of the C-terminal ends of A, B and C chains, this review makes arguments for the A-chain (ghA) to be most versatile module in terms of ligand binding (11).

C1q binding to the CH_2_ domain of antigen-bound IgG and subsequent classical pathway activation depends on its close proximity to the Fc region of adjacent IgG; C1q does not bind (or binds with very weak affinity to) circulating IgG monomers. Crucially, IgG CH_2_ domains contain Asp-297 N-linked glycan amenable to extension by terminal galactose and sialic acid residues. Using recombinant variants of CD20-specific monoclonal antibody, rituximab, Perschke et al. demonstrate that Fc-galactose enhances complement fixation, but only for IgG1 and IgG4, proposing a novel strategy to manipulate complement fixing ability of therapeutic antibodies. Following up on the C1q-IgG subclass interaction, Lilienthal et al. have examined the parallel between murine IgG1 and human IgG4 subclasses (both capable of inhibiting hexamerisation of IgG1 and IgG3 and subsequent C1q binding and classical pathway activation). The authors show that murine IgG1 suppresses IgG2a-mediated classical pathway activation. Since IgG subclass is of great importance in pathophysiology, galactose and sialic acid manipulation has therapeutic implications (Lilienthal et al.). It is worthwhile to note that allergen-based immunotherapy in allergic patients can give rise to increased IgG4 levels while dampening specific IgE production; thus, IgG4 subclass polarization also circumvents the classical pathways activation in allergen-desensitized patients (13). Moving on from immunoglobulin interaction of C1q to non-immune ligands, Donat et al. examined the implications of C1q binding to cholesterol crystals as well as von Willebrand factor (vWF) with respect to cholesterol crystal clearance by macrophages. Curiously, vWF bound cholesterol crystals via C1q, and the tripartite complex upregulated phagocytic and sensing receptors, such as MerTK, LRP-1, SR-A1, CD14, LAIR1, and PD-L1. vWF seems to interfere with the phagocytosis of cholesterol crystals and C1q complex. An assessment of pro-inflammatory cytokines revealed that vWF binding to C1q suppresses inflammatory response by macrophages, which may be relevant in atherosclerosis. El-Shamy et al. have reviewed the role of complement (and C1q) in chronic hepatitis C virus (HCV) infection and cryoglobulinemia since HCV-triggered complement activation is involved in liver fibrosis and type II cryoglobulinemia.

The last two papers, from Roberta Bulla group, in the volume allude to the fascinating involvement of C1q in tumor (Agostinis et al.; Mangogna et al.). C1q is expressed in the microenvironment of various types of human tumors, including melanoma, prostate, mesothelioma, and ovarian cancers. C1q promotes tumor by encouraging their adhesion, migration, and proliferation in addition to angiogenesis and metastasis (14). Agostinis et al. now report that C1q is found in good amounts in the tumor microenvironment of asbestos-induced malignant pleural mesothelioma (MPM), where it can interact with hyaluronic acid (HA), an abundant tumor microenvironment component. C1q-HA interaction seems to work in favor of MPM cells, suggesting that C1q can be exploited by tumor for its progression and invasion. In another study, Mangogna et al. have used Oncomine and UALCAN database in order to ascertain whether the transcriptional expression of the C1q three chains has a prognostic relevance for glioma. C1q is known to be expressed in the central nervous system and is considered a precipitation factor for neurodegeneration and neuroinflammation (9). The study reveals a positive correlation between higher levels of C1q expression and unfavorable prognosis in a diverse grade of gliomas, thus, giving a new dimension to C1q research. How C1q interacts with brain tumor cells as well as microglia and astrocytes in the context of gliomas needs further investigation.

In conclusion, C1q remains an ever important molecule of complement and innate immunity. Its versatility and modularity seem to offer enormous physiological potential; sometimes, it can be exploited by pathogens and tumor to their end. Future research in the area of cancer, pregnancy, aging and neuroinflammation is going to throw many pleasant surprises.

## Author Contributions

All authors listed have made a substantial, direct and intellectual contribution to the work, and approved it for publication.

### Conflict of Interest

The authors declare that the research was conducted in the absence of any commercial or financial relationships that could be construed as a potential conflict of interest.
